# The effect of community environment on the trajectory of depressive symptoms and cohort differences among middle-aged and older Chinese adults

**DOI:** 10.3389/fpubh.2024.1480295

**Published:** 2024-10-15

**Authors:** Xuyang Li

**Affiliations:** School of Public Health, Wuhan University, Wuhan, Hubei, China

**Keywords:** community environment, depressive symptoms, development trajectory, cohort differences, multi-level growth model

## Abstract

**Background:**

Urbanization has changed the living environment of middle-aged and older Chinese adults, but it also brings certain mental pressure to them. Few studies have explored the effect of community environment on the development trajectory and the cohort differences of depressive symptoms in Chinese individuals.

**Methods:**

Based on the longitudinal data of the China Health and Retirement Longitudinal Study (CHARLS) from 2011 to 2020, using three-level hierarchical linear growth model, this study examined the effect of community environment on the trajectory of depressive symptoms and cohort differences among middle-aged and older Chinese adults in five cohorts from 1920 to 1929, 1930–1939, 1940–1949, 1950–1959, and 1960–1966.

**Results:**

The results of this study showed that middle-aged and older adult individuals who lived in neighborhoods with better community physical and social environments had a protective effect on depressive symptoms. There was a cohort difference in the development of depressive symptoms among middle-aged and older Chinese adults. The baseline depressive symptoms in the later birth cohorts were higher than those in the earliest cohort, and the development rate were also significantly higher. The role of community environment in the development rate of depressive symptoms among middle-aged and older adult individuals varied across the cohort. A better community social environment had a more significant moderating effect on the development rate of depressive symptoms in the early birth cohort (1930–1939), and a better physical environment had a more significant moderating effect on the development rate of depressive symptoms in the 1940–1949 cohort.

**Conclusion:**

Under the strategic background of healthy aging in China, the construction and improvement of community environment should become an important part of coping with the realistic challenges of the aging population, such as the expanding scale of depressed population among the middle-aged and older adult individual.

## 1 Introduction

By the end of 2022, the population of China aged 45 or above accounted for 44.0% of the total population, while the population aged 65 or above accounted for 14.9% (1). With a large aging population, China is rapidly entering an aging society, which brings two main problems including older adult health problems. Depression is a common but serious psychological disorder that affects the older adults, peaking around the age of 50–60 (2). According to World Health Organization (WHO) data, more than 322 million people worldwide suffer from depression, accounting for 4.4% of the world’s population (3). Depression not only has a significant impact on the older adult emotions, quality of life and ability of daily activities (4), but also leads to an increase in medical expenses (5). The prevalence of depressive symptoms among the middle-aged and older Chinese adults ranges from 13 to 41% (6–10), while the rate of depression patients seeking medical attention in China is only 9.5% (11). Considering the background of China’s aging population worsening, the scale of the depressed middle-aged and the lack of older adult population being identified and treated, it is particularly important to study the factors related to depression in middle-aged and older Chinese adults and provide intervention evidence for preventing depression.

In China, with a persistent low fertility rate and massive population mobility, the traditional older adult care model relying mainly on families cannot be sustained. As the most important living and activity space for the older adults besides the family, the community is bound to assume more responsibilities for older adult care. Therefore, in light of the national conditions, the Chinese government has put forward some requirements for an older adult-friendly community, namely clean environment, accessible community services, complete and convenient travel facilities, extensive and sufficient social participation, a strong atmosphere of filial piety and respect for the older adults, etc. At the same time, WHO also calls for achieving an “age-friendly” society in eight areas: outdoor spaces and buildings, community support and health services, transportation, housing, communication and information, respect and inclusion, public participation and employment, and social participation. To sum up, the community environment can be mainly divided into two categories. One focuses on the physical environment, including the community infrastructure, how to build architectural environment to meet the different living needs of the older adults in the community and related design standards. The other focuses on the social environment, including the quality of social relationships of the older adults, and emphasizes the importance of the older adults participating in the community and realizing their own value (12). Accordingly, this study focuses on two aspects of community environment—physical environment and social environment.

Previous studies have shown that risk factors for depression include many individual characteristics, including gender, psychological factors (such as stressful life events, family history, etc.) (13). In contrast, there are less studies on the association between community environment and depression. However, there are many theoretical studies on neighborhood stressors to explain why community environment may be associated with residents’ mental health, especially depressive symptoms. On the one hand, the community environment such as lack of living facilities, inconvenient transportation, inadequate housing, and insufficient green space may become sources of stressors for individuals (14). On the other hand, community environment may affect social connections and the level of social support experienced by residents, which in turn may affect residents’ ability to resist stress and depressive symptoms (15). Therefore, the association between community environment and depression has attracted the attention of many public health and epidemiology experts (14, 16–18). Ivey’s study showed that neighborhood crime, unsafe traffic, and a community environment lacking neighborhood help were significantly positively associated with self-reported depressive symptoms (19). Soonhee found that people who were dissatisfied with their community environment were more likely to be depressed, indicating a strong correlation between perceived community security and depressive symptoms. The explanation is that individuals who feel unsafe or dissatisfied with the community environment may withdraw from social interactions (such as talking to neighbors or visiting friends, etc.) and lack appropriate health behaviors (such as outdoor sports, doctor visits, etc.) (20). Notably, some studies have shown that improvements in community environment may provide additional protection for “disadvantaged” older adults. For example, one study found that a community’s cultural and entertainment environment can significantly slow down the growth rate of depressive symptoms among the older adults (16). Another study found that the development of community cultural activities can also reduce the adverse impact of living alone on the mental health of the older adults (17).

Based on the results of existing studies, it can be found that cross-sectional data are mostly used, and it is unclear whether the community environment will have a sustained impact on the development of depressive symptoms in middle-aged and older adult individuals. Secondly, due to the limitations of the databases used, researchers usually use a single indicator (such as poverty rate and community socioeconomic status) or individual characteristic summaries (such as residents’ *per capita* years of education) to measure community environment, with less systematically examine the effects of different dimensions of community resources on mental health. The former often better reflects the level of regional economic development rather than being directly related to the living conditions of specific community residents; the latter, because it is highly correlated with individual characteristics, making it difficult to clarify the “net effect” of community characteristics on individual health outcomes (21). Furthermore, although most studies acknowledge the association between community environment and mental health, few studies have explored the heterogeneity of this association among different older populations. For example, a cross-sectional study in China reported that better community recreational (including basketball courts, swimming pools, outdoor fitness equipment, etc.) and supportive environments (including senior activity centers and senior associations) may reduce the risk of depression in middle-aged and older adults (22). Another cross-sectional study reported that location and traffic (including the number of buses/distance to bus stations), basic living (including sewer system, road material and road cleanliness, etc.), and older adult-friendly facilities environments (including post offices, convenience stores, supermarket, etc.) had significant positive effects on the mental health of rural community older adults, and the social interaction environment had positive effects on the mental health of urban older adults (23). A study using longitudinal data showed that good community infrastructure (including sewer system, waste management, libraries and outdoor exercise facilities, etc.) and organization (including dancing teams, family-based elder-care center, association of older adults, etc.) were significantly associated to a lower risk of depressive symptoms, and home nursing centers and outdoor sports facilities were significantly associated to a lower risk of depressive symptoms (24). In conclusion, it is not clear whether the association between “community environment-depression” is more significant in the earlier cohorts or the later cohorts. Therefore, it is of practical significance to study the cohort differences of community environment in the process of influencing individual depressive symptoms for intervention and guidance of existing health service system construction.

This study examined middle-aged and older adults born in five cohorts from 1920 to 1929, 1930–1939, 1940–1949, 1950–1959, and 1960–1966 in Chinese communities, using longitudinal data from 2011 to 2020 to measure individual depressive symptoms and reveal the changes in depressive symptoms within each cohort during the follow-up period, and explore the role of community environment in this process and possible cohort differences. Based on existing theories and empirical studies, we propose the following hypotheses: (1) Favorable community environment (including physical and social environment) has protective effects on baseline depressive symptoms and can slow the development of depressive symptoms in middle-aged and older adults. (2) There are significant cohort differences in the development of depressive symptoms among middle-aged and older adults in different cohorts. (3) The protective and moderating effects of favorable community environment on depressive symptoms and their development in middle-aged and older adults differ across cohorts.

## 2 Methods

### 2.1 Study design and participants

The study used data were extracted from the five rounds of follow-up surveys (2011, 2013, 2015, 2018, 2020) published by the China Health and Retirement Longitudinal Study (CHARLS), which includes high-quality microdata representing families and individuals aged 45 years and above in China, the CHARLS aimed to analyze the problem of population aging.

In this study, five cohorts of middle-aged and older adults interviewed in 1920–1929, 1930–1939, 1940–1949, 1950–1959, and 1960–1966 were selected from previous CHARLS surveys as analysis objects. The inclusion and exclusion criteria of this study were: (1) deleting the missing values of basic demographic characteristics (age, sex, education level, marital status, etc.); (2) delete samples with missing data due to reasons such as death or loss of follow-up during the follow-up investigation. Retain respondents who have completed the assessment of depressive symptoms in the initial interview survey and at least one follow-up survey; (3) most communities were missing only one or two community characteristic variables, and the missing rate for all community-levels variables was less than 2%. Therefore, samples with missing values on the community questionnaire were not removed in this study.

### 2.2 Exposure and outcome definition

#### 2.2.1 Assessment of outcome (depressive symptoms)

CHARLS used the simplified 10-item Version of Center for Epidemiological Studied Depression (CESD-10) to access the level of depressive symptoms in subjects. This scale focuses on individual emotional experience, including: (1) A total of eight negative emotional problems about whether the older adults are worried about small things, difficult to concentrate, depressed, laborious, fearful, restless, lonely and not get “going”; (2) The older adults are hopeful and happy about the future, a total of two positive emotional problems. Its effectiveness and reliability have been confirmed in the older adult population (25). All 10 questions of the scale were scored with four points (rarely or none of the time = 0, some or a little of the time = 1, occasionally or a moderate amount of time = 2, most or all of the time = 3), and the two positive emotional problems were scored in reverse. The total score of each item was accumulated to obtain the total score of individual CESD-10 (0–30 points). The higher the score, the more severe the depressive symptoms.

#### 2.2.2 Assessment of the independent variable (community environment)

Based on existing literature and data availability, this study focuses on two dimensions of community environment: community physical environment (built environment) and community social environment. The community physical environment score is the sum of six questions (ranging from 0 to 6), which mainly measure the state of community infrastructure (23, 26). The community was asked: counting affirmative responses on whether the community has paved roads as the main type of road, waste management, a sewer system, electricity 365 days throughout the year, indoor toilet as the main type of toilet, and public restroom. The community social environment score is the sum of three questions (ranging from 0 to 3 points) that measure voluntary organizations in the community and counting affirmative responses on whether the community has a calligraphy and painting association, the older adult association, and organizations for helping the older adults and the handicapped. These three categories represent different types of community support and services that have been shown to impact the health and well-being of older Chinese (14, 18, 27).

#### 2.2.3 Covariates

We controlled for sociodemographic and health characteristics at baseline that could help us better explain the relationship between community environment and depressive symptoms in middle-aged and older Chinese. The included sociodemographic characteristics were as follows: age, sex (male/female), marital status (married/unmarried or otherwise), education level (no formal education/yes), birth cohort (1920–1929/1930–1939/1940–1949/1950–1959/1960–1966), smoking states (yes/no), alcohol consumption (yes/no), sleep (≥7 h/ <7 h): according to the Healthy China Initiative (2019–2030), sufficient sleep was defined as ≥7 h per night, insufficient sleep was defined as <7 h per night (28), life satisfaction (from “extremely satisfied” to “extremely dissatisfied,” the total score ranges from 0 to 4 points), and family economic status (we took the logarithmic transformation of annual household income and divided it into four levels by quartile, namely “lower,” “middle-upper”, “middle-lower “and “higher”). Health characteristics include Body Mass Index (BMI, it is calculated by dividing the weight (kg) by the square of the height (m). Under normal circumstances, the BMI of Chinese adults is divided into normal group within 18.5–23.9 kg/m^2^, and the others are non-normal group), physical pain (yes/no), chronic diseases (divided into “none chronic diseases,” “one,” and “two or more”), self-rated health status (ranging from “excellent” to “poor,” with a total score range of 0–4 points), and activities of daily living (ADLs) (the ability of older individuals to take care of themselves in daily life, including bathing, dressing, going to the toilet, controlling defecation and urination, getting into and out of bed, and feeding themselves, those who cannot complete any one of these six daily activities independently were defined as disabled, with a total score range of 0–6 points). In addition, the attributes of the respondent’s community were included (urban/rural, according to the classification criteria of the National Bureau of Statistics), whether there are convenient medical institutions within the community (yes/no), and whether the community provides subsidies to people over 65 years old (yes/no).

### 2.3 Statistical analyses

In this study, a three-level hierarchical linear growth model was constructed for data analysis. The model fits the changes trajectory in the longitudinal data by estimating the initial values (intercepts) of individuals on the outcome variables and the slope of individuals over time, and can simultaneously consider the effect of variables from different levels on the trajectory and their cross-level interaction effects. The individual observational records serve as the first-level of analysis to explain the age-related process of depressive symptoms in the same individuals. The middle-aged and older adult individuals serve as the second-level of analysis to explore differences in depressive symptoms changes among individuals with different birth cohort characteristics (and other individuals characteristics). Communities serve as the third-level of analysis unit, used to examine the difference in depressive symptoms change process among individuals with different community characteristics. We take age as a fixed effect variable in the model, and include the quadratic term of age to reflect the nonlinear relationship between age and dependent variable. The intercepts at community level and individual level were used as random effects estimation parameters to reflect the inter-community and inter-individual differences in depressive symptoms, respectively. To account for the differences among different cohorts, we included the birth cohort and its interaction with age in the model. To consider the effect of community environment on depressive symptoms, we included the characteristics of community environment and its interaction with age in the model. In order to explore the cross-level interaction effect, we further introduced the tripartite interaction terms of birth cohort, community environment and age into the model. In addition, we control for other relevant characteristic variables at the individual and community levels in the model. We used the MIXED procedure in SAS Version 9.4 to estimate hierarchical linear models using maximum likelihood method.

The data analysis in this study was divided into five steps: (1) An unconditional mean model without any explanatory variables was constructed to test whether there was significant variation in depressive symptom scores at different levels. (2) Age and its quadratic term were introduced into the model as independent variables, and the pattern of depressive symptoms changing with age was determined according to the goodness of fit of the model. (3) Community environment and its interaction with age were included in the model to examine the effect of community environment on individual depressive symptoms and their change process. (4) The birth cohort and its interaction with age were included in the model to reveal the cohort differences in individual depressive symptoms and their change process. (5) The tripartite interaction term of birth cohort, community environment and age were further introduced to explore whether the effect of community environment on depressive symptoms and their changes has cohort differences.

## 3 Results

### 3.1 Baseline characteristics of participants

Table 1 provides relevant characteristic information of baseline samples. Most of the samples were female, and most of them were married. 44.50% of the sample had not received formal education, and nearly 50% of the family economic status was in the middle or lower level; nearly 70% of middle-aged and older adults suffer from chronic diseases. The 1920–1929 cohort had the highest mean age at first interview (84.48 years old) and the lowest proportion of people with formal education compared to the other four later-birth cohorts. The earlier birth cohort (1930–1939) had significantly higher ADLs than the other four cohorts (Mean = 0.61). There were significant differences in the above individual characteristics among different cohorts. The mean of community physical environment and social environment index were 2.79 (0–6) and 0.66 (0–3), respectively. The results of this study have shown that there are significant differences in the physical and social environmental characteristics of the communities where different birth cohorts live. We found that 80.20% of communities have convenient medical institutions, and only 23.30% of communities provide subsidies to people over 65 years old.

**Table 1:** Measurement and distribution of characteristic variables in different birth cohorts.

Variables	*N* (%)/Mean (SD)					
	1920–1929	1930–1939	1940–1949	1950–1959	1960–1966	Total
Community environment						
Physical environment (0–6)	3.30 (1.96)	3.10 (1.96)	2.74 (1.95)	2.75 (1.92)	2.79 (1.89)	2.79 (1.93)
Social environment (0–3)	0.72 (0.92)	0.80 (0.96)	0.64 (0.90)	0.65 (0.91)	0.65 (0.89)	0.66 (0.91)
Place of residence (urban): rural	77 (75.50)	633 (71.70)	1,993 (78.00)	3,133 (77.20)	2,046 (77.50)	7,882 (77.00)
Medical institutions (no): yes	81 (77.90)	7,033 (78.20)	2,107 (81.40)	3,267 (80.10)	2,124 (79.80)	8,282 (80.20)
Subsidies for people over 65 years old (no): yes	20 (19.60)	190 (21.20)	540 (21.00)	984 (24.10)	665 (25.00)	2,399 (23.30)
Individual characteristics						
Age	84.48 (2.43)	75.22 (2.60)	65.74 (2.84)	56.72 (2.72)	47.95 (1.78)	58.60 (8.97)
Sex (female): male	51 (49.00)	457 (50.70)	1,328 (51.00)	1,915 (46.60)	1,137 (42.40)	4,888 (47.00)
Educational level (no): yes	27 (26.00)	288 (32.00)	1,320 (50.70)	2,129 (51.80)	2,008 (74.90)	5,772 (55.50)
Marital status (unmarried and otherwise): married	46 (44.20)	583 (64.70)	2,140 (82.20)	3,636 (88.50)	2,389 (89.10)	8,794 (84.60)
Family economic status (lower):						
Middle-lower	18 (17.30)	187 (20.80)	709 (27.20)	1,126 (27.40)	623 (23.20)	2,663 (25.60)
Middle-upper	17 (16.30)	189 (21.00)	554 (21.30)	1,065 (25.90)	793 (29.60)	2,618 (25.20)
Higher	17 (16.30)	142 (15.80)	491 (18.90)	1,052 (25.60)	887 (33.10)	2,589 (24.90)
Smoking (no): yes	34 (32.70)	379 (42.10)	1,108 (42.60)	1,621 (39.50)	902 (33.60)	4,044 (38.90)
Alcohol consumption (no): yes	22 (21.20)	120 (13.30)	476 (18.30)	788 (19.20)	455 (17.00)	1,861 (17.90)
BMI (normal): non-normal	33 (37.10)	344 (44.70)	1,061 (46.10)	1,686 (47.00)	1,210 (52.20)	4,334 (47.80)
Sleep (≥7 h): <7 h	65 (62.50)	513 (56.90)	1,428 (54.90)	2,116 (51.50)	1,131 (42.20)	5,253 (50.50)
Physical pain (no): yes	29 (27.90)	291 (32.30)	923 (35.50)	1,431 (34.80)	786 (29.30)	3,460 (33.30)
Life satisfaction	1.83 (0.73)	1.84 (0.67)	1.89 (0.71)	1.96 (0.71)	2.00 (0.72)	1.94 (0.71)
ADLs	0.51 (1.18)	0.61 (1.23)	0.40 (0.98)	0.26 (0.80)	0.13 (0.57)	0.30 (0.86)
Self-rated health	2.91 (0.87)	2.94 (0.90)	2.97 (0.87)	2.87 (0.90)	2.71 (0.93)	2.86 (0.91)
Chronic diseases (none):						
One	22 (21.20)	238 (26.40)	765 (29.40)	1,205 (29.30)	817 (30.50)	3,047 (29.30)
Two and more	40 (38.50)	444 (49.30)	1,253 (48.20)	1,682 (40.90)	789 (29.40)	4,208 (40.50)

Reference group in parentheses.

There are significant differences between queues in the values of characteristic variables.

**Figure 1:**
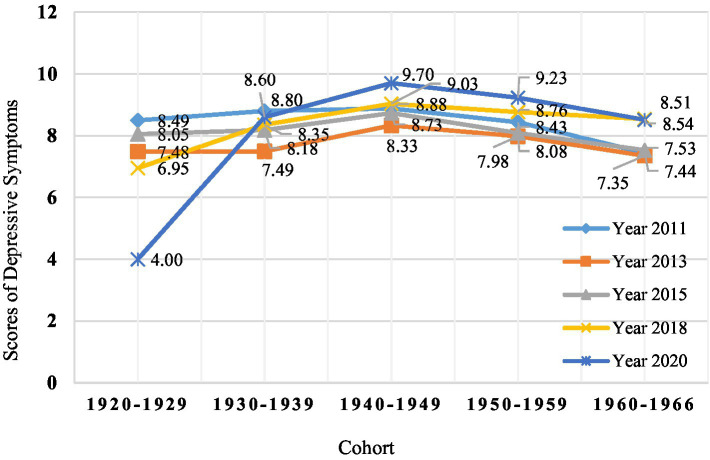
Depressive symptoms scores in five different birth cohorts in the CHARLS from 2011 to 2020.

Figure 1 shows the depressive symptom scores of each birth cohort at different observation points. At baseline, the average score for depressive symptoms across birth cohorts was 8.32. This score is consistent with previous reports of depressive symptoms in middle-aged and older adults using the same assessment tool (29–31). Over time, depressive symptoms in the later birth cohort increased to varying degrees. The scores of depressive symptoms in the earlier birth cohort were lower in recent years than those in the later birth cohort. The 1940–1949 cohort was higher than other birth cohorts at different time points. Based on previous studies on the trajectory of changes in the health level (including depressive symptoms) of middle-aged and older Chinese adults, this study suggests that the development trajectory of depressive symptoms in these five birth cohorts should be closer to a quadratic curve.

### 3.2 The effect of community environment on depressive symptoms in middle-aged and older adults

In this study, a three-level hierarchical linear growth model was constructed to fit the trajectory of depressive symptoms in middle-aged and older adult individuals. Model 1 in Table 2 established an unconditional mean model (empty model) based on depressive symptoms scores at different observation time points as the outcome variable. The intra-group correlation coefficient (ICC) calculated from the model estimates showed that age, individual and community could explain 42.60, 48.50, and 8.90% of the total variation in depressive symptoms, respectively. These results indicate that the three levels of factors have significant explanatory power for depressive symptoms in middle-aged and older adult individuals, so it is necessary to use a three-level hierarchical linear growth model for data analysis in this study.

**Table 2:** Results of multi-level growth model estimation of community environment on the trend of depressive symptoms in middle-aged and older adult people and their cohort differences.

	Model 1		Model 2		Model 3		Model 4		Model 5		Model 6		Model 7	
	Coefficient	SE	Coefficient	SE	Coefficient	SE	Coefficient	SE	Coefficient	SE	Coefficient	SE	Coefficient	SE
Intercept model														
Intercept	8.278*** (0.103)		8.478*** (0.107)		8.419*** (0.225)		8.416*** (0.225)		10.416*** (0.251)		11.348*** (0.306)		11.303*** (0.292)	
Age			0.065*** (0.005)		0.070*** (0.005)		0.070*** (0.005)		0.205*** (0.008)		0.394*** (0.032)		0.391*** (0.032)	
Age^2^			−0.002*** (0.000)		−0.002*** (0.000)		−0.002*** (0.000)		−0.001** (0.000)		0.007*** (0.001)		0.007*** (0.001)	
Community environment														
Physical environment					−0.334*** (0.045)		−0.335*** (0.045)		−0.302*** (0.044)		−0.303*** (0.044)		−0.467*** (0.114)	
Social environment					−0.193* (0.094)		−0.192* (0.094)		−0.162^+^ (0.092)		−0.160^+^ (0.092)		−0.462^+^ (0.246)	
Community environment × age														
Physical environment × age							−0.004 (0.003)		−0.002 (0.003)		−0.002 (0.003)		−0.017^+^ (0.009)	
Social environment × age							0.003 (0.007)		0.004 (0.007)		0.004 (0.007)		−0.018 (0.019)	
Cohort (Ref: 1960–1966)														
1950–1959									−1.797*** (0.132)		−2.863*** (0.241)		−2.824*** (0.241)	
1940–1949									−3.500*** (0.181)		−4.135*** (0.237)		−4.115*** (0.238)	
1930–1939									−5.741*** (0.274)		−3.897*** (0.546)		−3.728*** (0.548)	
1920–1929									−6.899*** (0.595)		2.478 (2.988)		2.421 (3.088)	
Cohort × age														
1950–1959 × Age											−0.158*** (0.030)		−0.156*** (0.030)	
1940–1949 × Age											−0.314*** (0.052)		−0.309*** (0.052)	
1930–1939 × Age											−0.508*** (0.080)		−0.518*** (0.080)	
1920–1929 × Age											−0.829*** (0.155)		−0.840*** (0.159)	
Cohort × community environment														
1950–1959 × Physical environment													0.159 (0.117)	
1940–1949 × Physical environment													0.032 (0.136)	
1930–1939 × Physical environment													0.259 (0.309)	
1920–1929 × Physical environment													1.148 (1.895)	
1950–1959 × Social environment													0.289 (0.253)	
1940–1949 × Social environment													0.422 (0.293)	
1930–1939 × Social environment													−1.613* (0.661)	
1920–1929 × Social environment													−1.221 (4.133)	
Cohort × community environment × age														
1950–1959 × Physical environment × age													0.007 (0.011)	
1940–1949 × Physical environment × age													0.025^+^ (0.013)	
1930–1939 × Physical environment × age													0.019 (0.019)	
1920–1929 × Physical environment × age													−0.003 (0.081)	
1950–1959 × Social environment × age													0.008 (0.024)	
1940–1949 × Social environment × age													0.009 (0.027)	
1930–1939 × Social environment × age													0.147*** (0.042)	
1920–1929 × Social environment × age													0.057 (0.174)	
**Var (community intercept)**	3.548 (0.310)		3.565 (0.316)		1.126 (0.135)		1.126 (0.135)		1.053 (0.128)		1.050 (0.128)		1.041 (0.126)	
**Var (individual intercept)**	15.782 (0.296)		15.655 (0.296)		8.950 (0.232)		8.953 (0.232)		8.743 (0.225)		8.736 (0.225)		8.842 (0.225)	
**Var (age intercept)**	20.523 (0.156)		20.237 (0.155)		20.308 (0.181)		20.305 (0.181)		20.081 (0.179)		20.057 (0.179)		20.115 (0.178)	
**AIC**	279,454.6		279,149.2		211,129.0		211,145.6		210,665.5		210,638.5		210,673.4	

The covariates included in Models 3–5 include gender, marital status, family economic status, number of chronic diseases, alcohol consumption, smoking, sleep, life satisfaction, physical pain, BMI, ADL, self-rated health at the individual level, as well as whether there are convenient medical institutions within the community and whether the community provides subsidies to people over 65 years old at the community level. Due to space limitations, the table cannot be presented.

***p < 0.001, **p < 0.01, *p < 0.05, +*p* < 0.1.

Each model presents unstandardized estimates and standard errors.

Based on model 1, model 2 included age as a fixed effect variable and included the quadratic term of age to reflect the non-linear trend of depressive symptoms. The results of this study showed that depressive symptoms of middle-aged and older adult individuals increased significantly with the age of the individuals (the coefficient of age first term was 0.065, *p* < 0.001). At the same time, the depressive symptoms of middle-aged and older adult individuals also showed a trend of first increasing and then decreasing with the increase of the number of visits (the coefficient of the quadratic term of age was −0.002, *p* < 0.001). We also tried to include other multiple terms of age, and compared the overall goodness of fit for models with different ages. The results showed that the model with both first and quadratic terms of age had better goodness of fit. Therefore, the subsequent models were based on Model 2 to fit the trend of depressive symptoms with age.

In order to reveal the effect of community environment on the change of depressive symptoms in middle-aged and older adult individuals, Model 3 and Model 4 included the community physical environment, the social environment and their interaction with age, respectively. The results showed that after controlling for individual characteristics and community-level structural characteristics, favorable community environment had a significant protective effect on the initial level of depressive symptoms in middle-aged and older adult individuals. Specifically, the better the physical environment of the community where they lived (coefficient = −0.334, *p* < 0.001), the lower the depressive symptoms score of middle-aged and older individuals. Similarly, the better the social environment of the community where they live (coefficient = −0.193, *p* < 0.05), the lower the depressive symptom score of middle-aged and older adult individuals, and the better the performance of depressive symptoms. It’s worth noting that we further explored the interaction terms between community physical environment and social environment with age, respectively, and found that there was no statistical difference in the interaction terms between community environment with age for depressive symptoms in middle-aged and older adults.

In addition, a series of sociodemographic and health characteristics (sex, marital status, family economic status, smoking, alcohol consumption, sleep, life satisfaction, BMI, ADLs, self-rated health, and chronic diseases) and community structural characteristics variables (convenient medical institutions and provide subsidies to people over 65 years old) were also included in models 3 and 4. The results of these control variables were consistent with the results of previous studies (4, 6, 8): depressive symptoms were more severe in women than in middle-aged and older adult men; the depressive symptoms of non-married group were higher than those of married. The lower of family economic status and the more types of chronic diseases, the more severe the depressive symptoms of the middle-aged and older adult groups; The depressive symptoms of groups with sleep duration <7 h were higher than ≥7 h; The depressive symptoms in the abnormal BMI group (wasting, overweight, obesity) were significantly higher than those in the normal group. In addition, the lower life satisfaction, the worse ADLs and self-rated health status, the more severe depressive symptoms of the middle-aged and older adult groups. In this study, we did not found that community-level variables such as the accessibility of medical facilities and the distribution of older adult allowances had a significant effect on depressive symptoms in middle-aged and older adults.

### 3.3 The effect of community environment on depressive symptoms in middle and older adults and cohort differences

Model 5 and Model 6 included the birth cohort and the interaction term with age, respectively. The results showed that, at baseline, depressive symptoms in the later birth cohort were significantly worse than those in the early cohort. The earlier birth cohort, the lower the baseline depressive symptoms score (coefficients for 1920–1929, 1930–1939, 1940–1949, and 1950–1959 cohort were −6.899, −5.741, −3.500, and −1.797, respectively, *p* < 0.001). More importantly, the interaction between the birth cohort and age in Model 6 was significant, indicating that the development rate of depressive symptoms among the middle-aged and older adults in different birth cohorts also showed significantly differences. The rate of development of depressive symptoms in the four groups of late birth cohort were significantly higher than that in the earliest birth cohort (coefficients for 1920–1929, 1930–1939, 1940–1949, and 1950–1959 cohort were −0.829, −0.508, −0.314, −0.158, respectively; *p* < 0.001).

To investigate whether there were cohort differences in the impact of community environment on depressive symptoms over time, Model 7 included cross-level interaction terms of community environment, birth cohort, and age on basis of Model 6. Firstly, we found that the regression coefficient of the cross-level interaction terms between community social environment, 1930–1939 cohort, and age was 0.147 (*p* < 0.001). This suggests that the improvement of community social environment has a more significant moderating effect on the development rate of depressive symptoms in the earlier birth cohort. In other words, although improvements in community social environment can significantly slow down the development speed of depressive symptoms in different birth cohorts, the effect was weaker in later birth cohort. The regression coefficient of the cross-level interaction terms between community physical environment, 1940–1949 birth cohort, and age was 0.025 (*p* < 0.1), indicating that the improvement of community physical environment had a more significant moderating effect on the development rate of depressive symptoms in this birth cohort. Additionally, the regression coefficient of interaction terms between community social environment and 1930–1939 birth cohort was −1.613 (*p* < 0.05). This suggests that favorable social environment had a weaker protective effect on depressive symptoms in the 1930–1939 cohort compared with the reference group. The results of this study showed that no significant results were found in the interaction terms between community physical environment and each birth cohort.

## 4 Discussion

This study used a three-level hierarchical linear growth model to examine the effect of community environment on the development trajectory of depressive symptoms in middle-aged and older adults and five cohorts differences between 1920 and 1966. This study first confirmed hypothesis 1, that a favorable community environment has a protective effect on depressive symptoms in middle-aged and older adults, consistent with previous results (32, 33). This finding supports the collective resource theory that individuals living in areas with more and better collective social and physical resources are healthier than other groups (34). For example, increasing the number of sidewalks and parks in neighborhoods is significantly associated with depression in older adults (33, 35, 36), and better community walkability has been shown to lower the risk of depression in older men (33). Poor basic living facilities, such as fragile sewer systems, lowers people’s subjective well-being (37), which in turn affects residents’ mental health. The lack of affordable public transportation in communities restricts individuals’ participation in social activities, thereby increasing social isolation and loneliness (38). Existing recreational facilities not only directly promote outdoor activities of the older adults, but also indirectly affect their physical and mental health (39). Social support and participation play a crucial role in regulating depressive symptoms by improving community attachment (40). Therefore, it is suggested that in order to improve the aging of the community where the middle-aged and the older adults live, we should not only focus on the built environment of the community, but also on the construction of social leisure space and cultural atmosphere, carry out diversified cultural entertainment and group activities that meet the preferences and needs of the older adults, and give play to the functional role of the community environment in enhancing the social interaction and mental health of the older adults.

Another important finding of this study is that there is a significant cohort difference in the development of depressive symptoms among middle-aged and older adult people in China, which also confirmed hypothesis 2 mentioned above. The scores of depressive symptoms in the later birth cohorts were significantly higher than that in the earliest cohort, and the development rate of depressive symptoms in the later birth cohorts were faster. The analysis of the mental health development trajectory of the older adults by Gao (41), Sullivan (42) and other scholars also showed similar results. The reasons for this may be growing economic uncertainty, accelerated pace of modern life, and the increasing stress leading to anxiety, disappointment and distress, which can increase depression levels in younger cohorts (43, 44). In addition, a culture of individualism and consumption that emphasizes extrinsic values (such as money and fame) also offers some explanations for the observed mostly rising trends in depressive symptoms (44). On the basis of considering the effect of previous individual factors such as social and economic status, health status and behavior on depressive symptoms in middle-aged and older adults (45, 46), this study also focused on the effect of community environment on depressive symptoms and their development rate among middle-aged and older adults. The results of this study showed that favorable community physical and social environment had a positive protective effect on the baseline depressive symptoms of the older adults in different cohorts, which is consistent with the results of most previous domestic and foreign studies on environment and depressive symptoms in middle-aged and older adults (24, 47–49). Although there are differences in physical health status, behavior pattern and family environment among the older adults in different birth groups, the protective effect of improving the level of community physical and social resources on depressive symptoms is stable. So, increasing the supply of various community resources and improving the community ecology and interpersonal environment should become the key work in the process of further improving the “community-based” older adult care service system.

The study further found that with age, the effect of community environment on the development rate of depressive symptoms was significantly different among middle-aged and older adults in different birth cohorts. For example, although a favorable community social environment had a moderating effect on the development of depression in different birth cohorts, the effect was most pronounced in the earlier birth cohorts (1930–1939). We speculate that middle-aged people, limited by their own work or important roles in the family, such as raising children, usually do not have leisure time to participate in community activities and organizations, and are not as active as older people, and generally do not benefit directly from the recreational activities and social organization activities currently carried out in most communities (50). Accordingly, their use rate of the community public facilities (such as activity centers for the older adults, etc.) is usually low. However, it is gratifying that we also found that the improvements of community physical environment had a more significant mitigating effect on the development rate of depressive symptoms in the 1940–1949 birth cohort compared to other birth cohorts. This may be because the supply of community physical resources can directly affect many aspects of the daily life and health needs of target population (such as transportation, healthcare, etc.), thereby producing more positive effects on protecting the mental health of older adult people (51). Given that the older adult population is already a high-risk group for mental problems, future research can explore in more detail which specific community environmental improvement measures are helpful in maintaining the mental health level of the older adult population.

This study conducted a systematic study on the effect of community environment on depression symptoms and cohort differences in middle-aged and older adults, which provides a basis for improving mental health policies in China. First of all, community infrastructure should be further improved, such as adding and improving the sewer system, garbage disposal methods, basic fitness facilities, etc., so that the community environment in the middle-aged and older adult groups to play more health benefits, while pleasing the body and mind, expand interpersonal social circle, so as to link the inner negative emotions (23). Secondly, we should pay more attention to the spiritual and cultural construction in the community environment, and increase the community recreational activities can effectively improve the psychological condition of the older adults. During the community planning and redevelopment phase, local government officials or community managers should consider implementing design and land use policies that support the prioritization of recreational facilities in community buildings (24). Finally, while considering the improvement of the physical and social environment of the community, it is necessary to pay attention to the subjective initiative and social participation of the middle-aged and the older adults, especially the middle-aged group. Through multiple channels to encourage them to actively participate in social activities and outdoor sports, make full use of community resources to improve physical and mental quality, prevent and control the occurrence of depressive symptoms. But there are still the following limitations. First, because the information about the community environment in the CHARLS data was collected only in the baseline survey, this study could not examine the effect of changes in the characteristics of the community environment on the middle-aged and older adults over time. Secondly, this study used the five longitudinal data published by CHARLS, and future studies should use more longitudinal tracking data to build a growth curve model with better fitting effect, so as to better reveal the specific pattern of the change trajectory of depressive symptoms. In addition, while CESD-10 is a commonly used tool for measuring depressive symptoms, it is still based on a self-reported score, which poses a threat of information bias. Therefore, more studies are needed in the future to more precisely explore the relationship between community environment and medically diagnosed depressive symptoms.

## 5 Conclusion

In summary, this study used longitudinal tracking data and a three-level hierarchical linear growth model to examine the effect of community environment on depressive symptoms and their change process, which filling the gap in previous studies that used cross-sectional data in examining the association and direction of the relationship between environment and depressive symptoms. This study revealed the crucial effect of community environment on the development of depressive symptoms among middle-aged and older Chinese adults, as well as the difference of the effect among different birth cohorts. It provides empirical evidence for developing more targeted policies and interventions to improve the mental health of middle-aged and older adults, and creating social support and living environments conducive to maintaining mental health will help reduce the disease risk in an aging society.

## Data Availability

Publicly available datasets were analyzed in this study. This data can be found at: https://charls.charlsdata.com/index/zh-cn.html.
